# Effect of Virtual Reality Technology on Attention and Motor Ability in Children With Attention-Deficit/Hyperactivity Disorder: Systematic Review and Meta-Analysis

**DOI:** 10.2196/56918

**Published:** 2024-11-27

**Authors:** Chuanwen Yu, Cheng Wang, Qi Xie, Chaoxin Wang

**Affiliations:** 1 School of Physical Education and Health, Heze University, Mudan District, Heze City, Shandong Province, China; 2 School of Physical Education, Baoji University of Arts and Sciences, Baoji City, China

**Keywords:** virtual reality, VR, immersive technology, attention deficit hyperactivity disorder, ADHD, hyperactivity, attention deficit, neurodevelopment, neurodevelopmental disorder, attention, motor ability, virtual reality technology

## Abstract

**Background:**

Attention-deficit/hyperactivity disorder (ADHD) is one of the common neurodevelopmental disorders in children and virtual reality (VR) has been used in the diagnosis and treatment of ADHD.

**Objective:**

This paper aims to systematically evaluate the effect of VR technology on the attention and motor ability of children with ADHD.

**Methods:**

The intervention method of the experimental group was VR technology, while the control group adopted non-VR technology. The population was children with ADHD. The outcome indicators were attention and motor abilities. The experimental design was randomized controlled trial. Two researchers independently searched PubMed, Cochrane Library, Web of Science, and Embase for randomized controlled trials related to the effect of VR technology on ADHD children’s attention and motor ability. The retrieval date was from the establishment of each database to January 4, 2023. The PEDro scale was used to evaluate the quality of the included literature. Stata (version 17.0; StataCorp LLC) was used for effect size combination, forest map-making, subgroup analyses, sensitivity analyses, and publication bias. GRADEpro (McMaster University and Evidence Prime Inc) was used to evaluate the level of evidence quality.

**Results:**

A total of 9 literature involving 370 children with ADHD were included. VR technology can improve ADHD children’s attention (Cohen *d*=−0.68, 95% CI −1.12 to −0.24; *P*<.001) and motor ability (Cohen *d*=0.48, 95% CI 0.16-0.80; *P*<.001). The intervention method and diagnosis type for VR technology had a moderating effect on the intervention’ impact on children’s attention (*P*<.05). The improvement in children’s attention by “immersive” VR technology was statistically significant (Cohen *d*=−1.05, 95% CI −1.76 to −0.34; *P*=.004). The improvement of children’s attention by “nonimmersive” VR technology was statistically significant (Cohen *d*=−0.28, 95% CI −0.55 to −0.01; *P*=.04). VR technology had beneficial effects on both children with an “informal diagnosis” (Cohen *d*=−1.47, 95% CI −2.35 to −0.59; *P*=.001) and those with a “formal diagnosis” (Cohen *d*=−0.44, 95% CI −0.85 to −0.03; *P*=.03).

**Conclusions:**

VR technology can improve attention and motor ability in children with ADHD. Immersive VR technology has the best attention improvement effect for informally diagnosed children with ADHD.

## Introduction

Attention-deficit/hyperactivity disorder (ADHD) is one of the most common neurodevelopmental disorders in childhood, featuring inattention, hyperactivity, or impulsivity that is not commensurate with age and development level [1]. Epidemiology shows that 5% of children worldwide endure ADHD, and the prevalence rate shows an upward trend. ADHD has become one of the important problems in the field of children’s mental health [2,3]. In addition, 45%‐70% of children with ADHD still have motor ability problems. They often show clumsiness and discordance in daily life, which affects their performance in learning and motor activities, and has adverse effects on their social ability, peer relationships, and physical and mental health [4,5].

As an integrated technology, virtual reality (VR) technology can enable participants to interact with things in the virtual world in real-time through realistic 3D vision, hearing, touch, and other forms [6,7]. Serious games are a form of video games that are not purely for entertainment but are often employed in fields such as education and medicine for learning or problem-solving purposes [8]. Serious games emphasize the integration of educational elements, with the goal of specifically enhancing certain abilities or skills. VR technology provides dynamic and realistic new social contexts for serious games, enriching their content. For instance, engaging in activities such as role-playing as a dolphin trainer or learning a musical instrument within a VR environment encourages patients to actively explore and experience scenarios [9,10], thereby improving attention and social motivation. VR technology has many advantages in the field of children patients, such as simulating their daily living environment, independently evaluating the influence of interference factors, and promoting the change of children’s cognition and behavior [11]. At present, a multitude number of studies have shown that VR technology can be used to evaluate children with ADHD [12-17]. In addition, VR technology is also helpful for the rehabilitation of children with ADHD [18-20]. Moreover, serious games based on VR technology are also beneficial for the rehabilitation of children with ADHD. Schena et al [21] conducted a VR game intervention for children with ADHD for 6 months and found that the hyperactivity, conflict, and executive function of children were significantly improved. Weerdmeester et al [22] conducted VR games for children with ADHD for 3 weeks. The results also found that compared with the control group, VR games had more advantages in improving ADHD children’s attention and motor ability. In addition, Frolli et al [23] used VR technology to learn the history of children with ADHD for 6 months, while the control group used traditional history learning methods. The results showed that the learning improvement of children with VR technology was more obvious.

By studying the previous literature, we found that there were meta-analyses to explore the intervention effect of VR technology on children with ADHD. Corrigan et al [24] conducted a meta-analysis of seven studies and found that immersive VR technology can improve ADHD children’s attention, but did not further clarify the “dose-response relationship” between intervention factors and attention improvement effect. A meta-analysis by Romero-Ayuso et al [25], which included 4 studies, found that VR technology had selectivity and specificity in improving attention in children with ADHD. In addition, there is no systematic study to evaluate the effect of VR training on the motor ability of children with ADHD. On this basis, this paper added new evidence to further clarify the “dose-response relationship” between intervention factors and attention improvement effect. Besides, we also increased the outcome indicators of motor ability, and discussed the impact of VR technology on children’s motor ability, to provide evidence for clinical practice and theoretical reference for researchers.

## Methods

### Research Framework

Based on the International Classification of Functioning, Disability and Health classification system and framework, this study followed the methods and requirements of the PRISMA (Preferred Reporting Items for Systematic Reviews and Meta-Analyses) statement and Cochrane Workbook [26,27]. The research plan for systematically evaluating the impact of VR technology on ADHD children’s attention and motor ability has been registered on international system evaluation platform PROSPERO [28] (crd42024499199). The PICO (Patient; Intervention; Comparison, Control, or Comparator; and Outcomes) framework of this study is shown in Multimedia Appendix 1.

### Literature Retrieval Strategy

Two researchers independently searched PubMed, Embase, Cochrane Library, and Web of Science databases for randomized controlled trials (RCTs) of VR technology on children with ADHD. The retrieval date was from the establishment of each database to January 4, 2023. The retrieval method adopted the combination of subject words and free words, and used the Boolean operation symbols “AND” and “OR” for combination connection, which is determined after repeated preinspection. If 2 researchers encountered disagreements, a third researcher would join in the discussion and make joint decision. A subsequent supplement was conducted to trace relevant systematic reviews and references of included papers for those not having been retrieved, and the specific retrieval strategy is shown in Multimedia Appendix file. See Multimedia Appendix 2 for specific retrieval strategies.

### Inclusion and Exclusion Criteria

Inclusion criteria were children (aged 5‐18 years) who were formally diagnosed with ADHD and met the *Diagnostic and Statistical Manual of Mental Disorders* (*DSM-V*, *DSM-IV*, and *DSM-IV-TR*) or *ICD-10* (*International Statistical Classification of Diseases, Tenth Revision*); people with ADHD diagnosed without formal diagnosis, but showed ADHD symptoms, such as inattention and hyperactivity disorder observed by outsiders. The intervention group used VR technology, while the control group did not use it. The outcome indicators meant the outcome indicators of attention and motor ability. If there were two or more data measuring attention at the same time in an included article, we would select the data in the article that has the highest use of frequency in other included articles. The outcome indicators of attention include: continuous performance test, Stroop color, Advanced Test of Attention, visual search task, and Go-NoGo. Exercise capacity includes: graphomotor testing, the Movement Assessment Battery for Children, and German Motor Test.

Exclusion criteria were outcome indicators were inconsistent or data could not be extracted, literature could not found; nonrandomized controlled trial, or repeatedly published or poorly evaluated literature.

### Literature Screening

Two researchers independently screened the literature according to the inclusion and exclusion criteria. First, the retrieved literature were imported into EndNote X9 (Clarivate) to eliminate duplicate literature and read the titles and abstracts of the literature for preliminary screening. Second, the full-text reading of the screened literature was conducted for rescreening to determine the final included literature. If two researchers encountered disagreements, a third researcher would join in the discussion and make joint decision.

### Data Extraction

Two researchers extracted data independently. The extracted information includes basic information (author, year of publication, age, sample size, or diagnosis information), intervention characteristics (intervention method, intervention duration, intervention cycle, and intervention frequency), and outcome indicators. If the data was missing or unclear, the original author would be contacted through email. If 2 researchers extracted different information, a third researcher would join in and made a joint decision.

### Literature Quality Evaluation

Two researchers independently used PEDro scale to evaluate the quality of literature [29]. In case of disagreement, a third researcher would join in and made a joint decision. The scale includes 10 items, such as “random allocation,” “allocation concealment,” “baseline similarity,” “blinding of participants,” “blinding of therapists,” “blinding of outcome assessment,” “participation rate >85%,” “intention-to-treat (ITT) analysis,” “intergroup analysis of statistical results,” and “point measurement difference.” Score standard: 1 point for meeting a certain standard; 0 point for not meeting the standard. The total score of the scale was 10 points, <4 points meant low quality, 4-5 points meant medium quality, 6-8 points meant good quality, and 9-10 points meant high quality. This paper only included literature with medium quality or above.

### Evaluation of Outcome Evidence

GRADEPro software was used to evaluate the grade of outcome evidence [30]. There are 5 evaluation items, including limitations, inconsistencies, indirectness, imprecision, and publication bias. Included literature were evaluated one by one (none [not degraded], serious [1 grade reduced], very serious [2 grades reduced]). Evidence was marked as 4 levels: high quality, medium quality, low quality, and very low quality. The results were presented in the evidence summary table. “High”: very confident that the predicted value was close to the real value; “Medium”: moderate confidence in the predicted value, which may either be close to the real or differ greatly; “Low”: the predicted value was limited, which may be very different from the real value; “Extremely low”: the predicted value was almost uncertain, and there was likely to be a big difference between the predicted value and the real value. The level of evidence is evaluated by two researchers independently. If two researchers encountered disagreements, a third researcher would join in the discussion and make joint decisions.

### Statistical Analysis

The evaluation data extracted in this study were all change values (posttest data minus pretest data or the data in the intervention minus the pretest data), and the extraction form was mean (SD). Stata (version 17.0) was used for effect size combination, subgroup analysis, sensitivity analysis, and publication bias. Due to the use of different evaluation tools, standard mean difference was selected for effect size combination, and point estimation and 95% CI were given. When *P*<.05, the difference was statistically significant. Cohen *d* was selected as the effect size, and Hedges [31] and Olkin were used to correct the standard error to calculate the effect size, *P*<.05 was statistically significant, <0.20 indicated a small effect; 0.2‐0.49, a small-to-moderate effect; 0.50‐0.79, a medium effect; and ≥0.80, a large effect [32]. If the measurement unit included in the measurement tools had different directions, multiply by −1 to ensure that the directions of the units were consistent. Heterogeneity used Higgins’ *I*^2^ statistics [33]. It was divided into low (25%), medium (50%), and high (75%) heterogeneity. If there was heterogeneity, the random effect model would be used to merge the effect size, and the source of heterogeneity would be discussed through subgroup analysis and sensitivity analysis. Otherwise, the fixed effects model would be adopted.

## Results

### Literature Search Results

**Figure 1.**
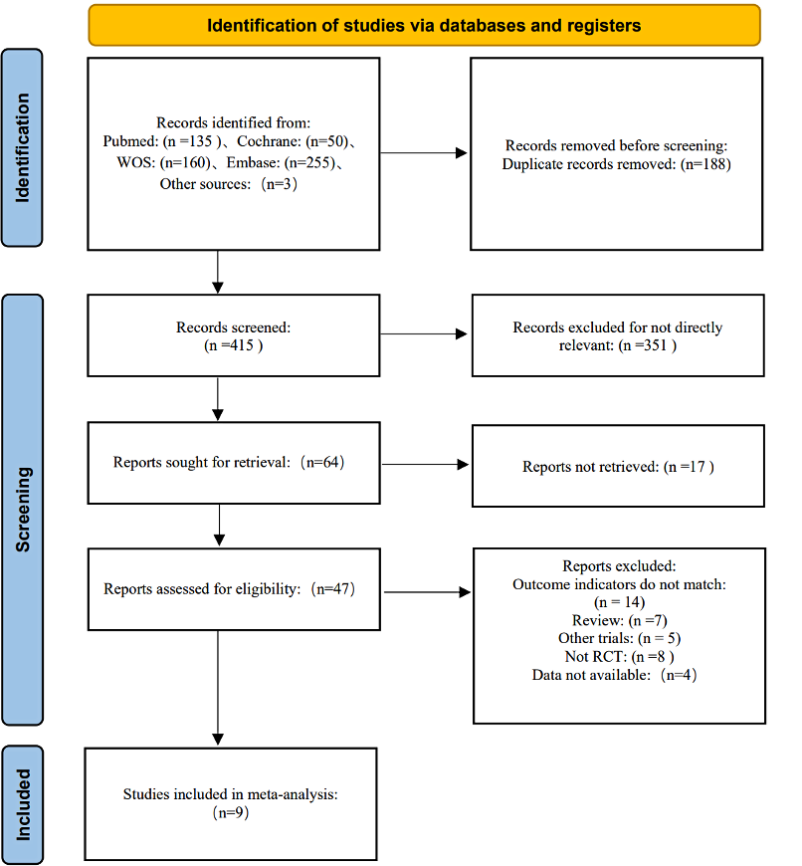
Flowchart of literature screening. RCT: randomized controlled trial; WOS: Web of Science.

The two researchers obtained 603 articles, among which 135 were from PubMed, 160 were from Cochrane, 50 were from Web of Science, 255 were from Embase, and 3 were from other sources. EndNote X9 has 415 articles remaining after removing 188 duplicate articles. Then, after reading the title and abstract, 351 irrelevant articles were excluded, thus leaving 64 articles. Among them, 17 articles cannot be found. Therefore, full-text reading was conducted on the final 47 articles, among which there were 14 articles having inconsistent outcome indicators, 7 review articles, 5 other studies, 8 nonrandomized controlled trial studies, and 4 articles that we were unable to obtain data for. As a result, a total of 9 articles were included [22,34-41], see Figure 1.

### Characteristics of the Included Literature

A total of 9 articles were included (Table 1), published from 2001 to 2022, with a sample size of 201 in the intervention group and 169 in the control group, aged 7‐15 years. The authors of the literature come from France, China, Republic of Korea, the Netherlands, Poland, and Switzerland. The intervention methods of the intervention group included VR games, VR cognitive training, and VR neural feedback training. The intervention methods for the control group included drug therapy, conventional therapy, and traditional neural feedback training. The intervention duration lasted 10‐60 minutes, the intervention cycle was 2‐12 weeks, and the intervention frequency was not indicated in some studies, while in other studies it was 1‐4 times per week. The outcome indicators of attention were continuous performance test–omission, Advanced Test of Attention–omission, visual search task–reaction time, Go-NoGo false alarms and span backward task, and the outcome indicators of motor ability were Movement Assessment Battery for Children–Fine motor skills, German Motor Test–total and graphomotor testing–mean stroke velocity.

**Table 1.** Characteristics of included literature.

Included literature	Sample size (E/C) or (E1/E2/C)^a^^,^^b^	Diagnostic type	Country	Age (years), mean (SD) (E/C) or (E1/E2/C)	Type of intervention	Intervention characteristics	Outcome
Bioulac et al (2018) [20]	16/16	*DSM-IV*/^c^ formal diagnosis	France	9.5 (1.2)/ 8.4 (0.99)	E: virtual classroom cognitive remediation C: methylphenidate	30 min, 2 times/wk, 6 wk	CPT-omissions^d^
Chang et al (2022) [36]	16/16	*DSM-IV*/ formal diagnosis	China	8.38 (1.2)/ 8.38 (1.31)	E: VR^e^ games C: conventional therapy	60 min, 3 times/wk, 12 wk	Stroop color, GFT-mean^f^ stroke velocity
Skalski et al (2021) [37]	28/29/30	*DSM-IV*/ formal diagnosis	Poland	13.29 (1.55)/ 12.38 (1.70)/ 12.60 (1.61)	E: Unlimited neurofeedback training in VR(A) environments or neurofeedback training in a limited VR(B) environment: C: Traditional neural traditional feedback training	30 min, 1 times/wk, 10 wk	VST-^g^ reaction time
Cho et al (2004) [39]	9/9	The participants had ADHD^h^ symptoms; informal diagnosis	Republic of Korea	14‐18	E: Neurofeedback training in VR environments; C: no intervention	20 min, 4 times/wk, 2 wk	CPT- omissions
Kim et al (2020) [40]	20/20	Psychological diagnosis or formal diagnosis	Republic of Korea	8‐10	E: VR games; C: no intervention	30 min, 6 wk	ATA-omission^i^
Cho et al (2002) [38]	8/9	The participants had ADHD symptoms; informal diagnosis	Republic of Korea	13 (0.82)/14.67 (0.5)	E: VR cognitive training; C: neurofeedback training	20 min, 4 times/wk, 2 wk	CPT (omission）
Lee et al (2001) [41]	10/10	The participants had ADHD symptoms; informal diagnosis	Republic of Korea	—^j^	E: VR neurofeedback training; C: no intervention	10 min, 2 wk	CPT (omission）
Weerdmeester et al (2016) [22]	37/36	VragenLijst; formal diagnosis	Netherlands	9.84 (1.71)/9.69 (1.79)	E: VR games C: conventional therapy	15 min, 2 times/wk, 3 wk	Go-NoGo- false alarms, MABC-2-NL-^k^ fine motor skill
Benzing and Schmidt (2019) [34]	28/23	*ICD-10;*^l^ formal diagnosis	Switzerland	10.46 (1.30)/ 10.39 (1.44)	E: VR games C: conventional therapy	30 min, 3 times/wk, 8 wk	Span backward task or GMT-total^m^

a E: experimental group.

b C: control group.

c *DSM-IV*: *Diagnostic and Statistical Manual of Mental Disorders* (Fourth Edition).

d CPT: continuous performance test.

e VR: virtual reality.

f GFT: graphomotor function test.

g VST: visual search task.

h ADHD: attention-deficit/hyperactivity disorder.

i ATA: Advanced Test of Attention.

j Not available.

k MABC-2-NL: Movement Assessment Battery for Children.

l *ICD-10*: *International Classification of Diseases, Tenth Revision*.

m GMT: German Motor Test.

### Literature Quality Evaluation

The 9 included literature all achieved “random allocation,” “baseline similarity,” “withdrawal rate <15%,” “statistical analysis between groups,” and “point measurement and variation value.” Only 2 papers achieved “distribution hiding,” 2 papers achieved “blinding of research objects,” 1 paper achieved “blinding of evaluation,” 1 paper did not achieve “intention-to-treat (ITT) analysis,” and all papers did not achieve “blinding of therapists.” Further, 6 articles scored 6 points; 2 articles scored 7 points; 1 article scored 8 points. The average score of the 9 articles was 6.44 (SD 0.68), showing relatively good quality, as shown in Table 2.

**Table 2.** Methodological quality assessment of included literature.

Included literature	1^a^	2^b^	3^c^	4^d^	5^e^	6^f^	7^g^	8^h^	9^i^	10^j^	TS^k^
Bioulac et al (2018) [20]	1	0	1	0	0	0	1	1	1	1	6
Chang et al (2022) [36]	1	1	1	0	0	1	1	1	1	1	8
Skalski et al (2021) [37]	1	0	1	0	0	0	1	1	1	1	6
Cho et al (2004) [39]	1	0	1	0	0	0	1	1	1	1	6
Kim et al (2020) [40]	1	0	1	0	0	0	1	1	1	1	6
Cho et al (2002) [38]	1	0	1	0	0	0	1	1	1	1	6
Lee et al (2001) [41]	1	0	1	0	0	0	1	1	1	1	6
Weerdmeester et al (2016) [22]	1	1	1	1	0	0	1	0	1	1	7
Benzing and Schmidt (2019) [34]	1	0	1	1	0	0	1	1	1	1	7

a 1: allocation of randomization.

b 2: allocation concealment.

c 3: similarity baseline.

d 4: participants were blinded.

e 5: blinding of therapist.

f 6: assessor blinding.

g 7: more than 85% retention.

h 8: intention-to-treat (ITT) analysis.

i 9: between-group comparisons.

j 10: point and variability measures.

k TS: total score.

### Meta-Analysis Results

#### Meta-Analysis of the Effect of VR Technology on Attention in Children With ADHD

**Figure 2.**
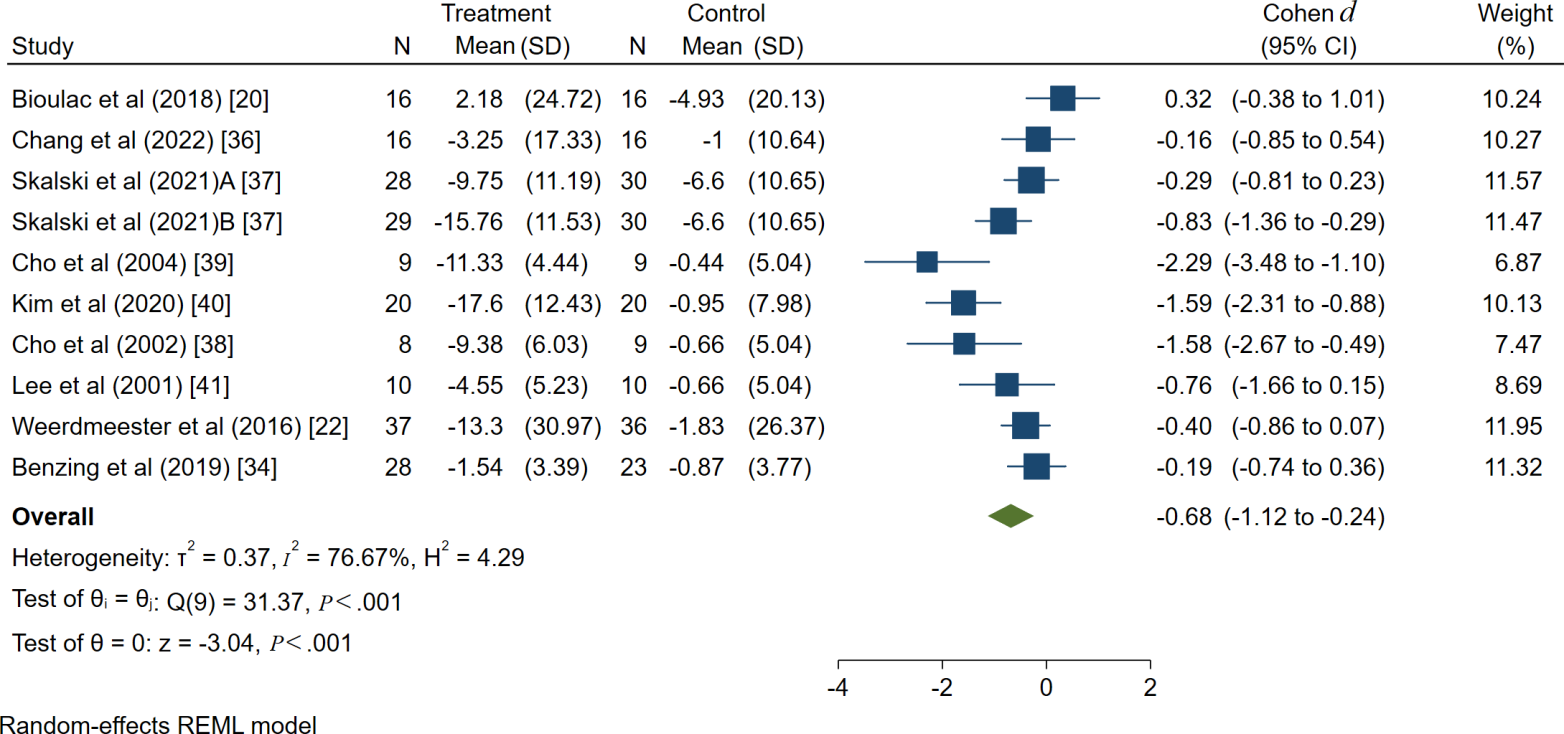
Forest diagram of the effect of virtual reality technology on attention of patients with attention-deficit/hyperactivity disorder. REML: restricted maximum likelihood.

A total of 9 literature covering 10 study projects and 370 patients discussed the intervention effect of VR technology on the attention of patients with ADHD. Heterogeneity analysis revealed an *I*^2^ value of 76.11%, indicating moderate heterogeneity. Hence, the random effect model was used for effect size combination. Meta results revealed a Cohen *d* of −0.68 (95% CI −1.12 to −0.24, *P*<.001) and the difference was statistically significant, indicating that VR technology could improve the attention of patients with ADHD, as shown in Figure 2.

**Figure 3.**
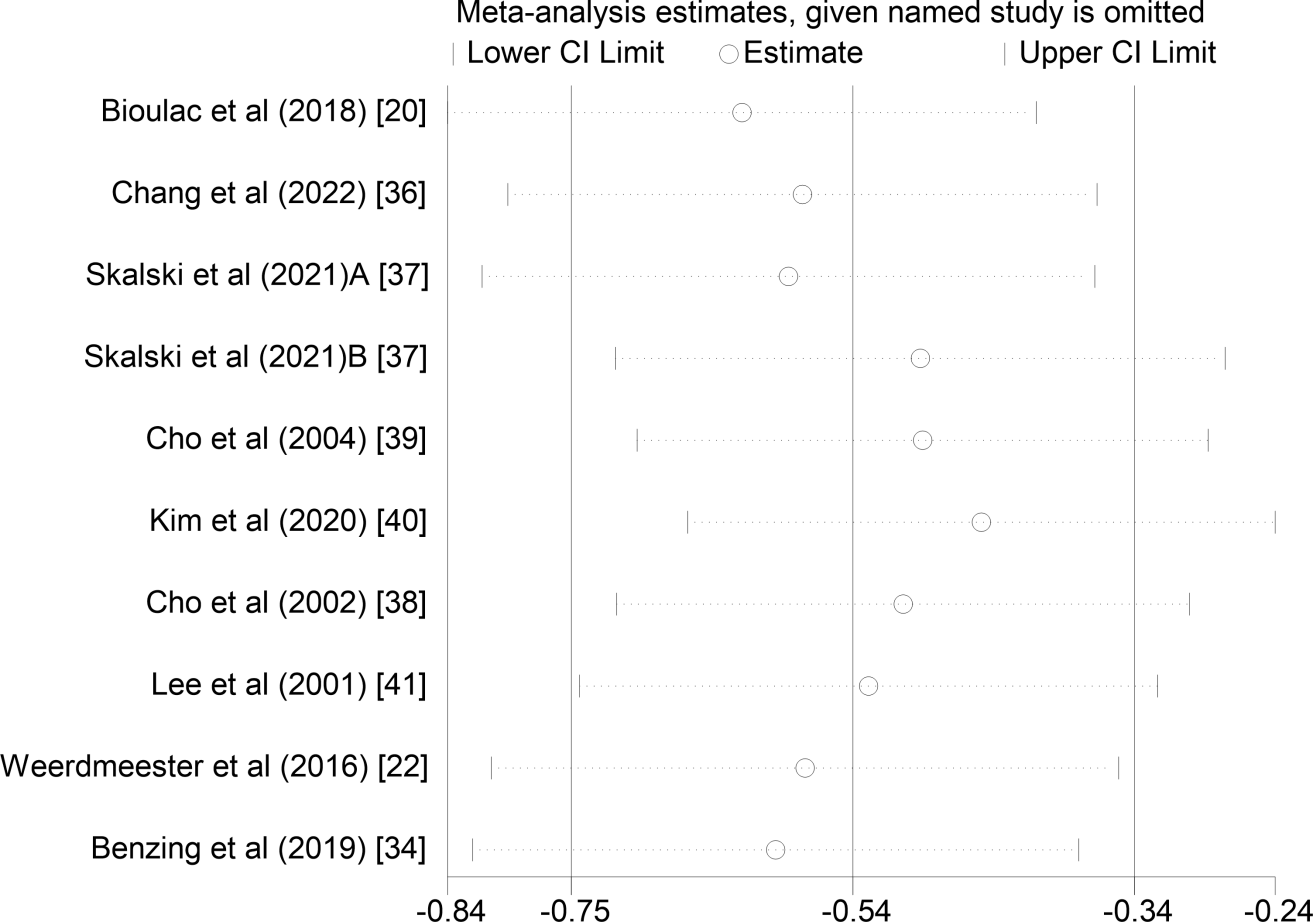
Sensitivity analysis of the effect of virtual reality technology on attention of patients with attention-deficit/hyperactivity disorder.

To explore whether the heterogeneity between studies is caused by a single study, sensitivity analysis was performed using Stata (version 17.0). After eliminating each study one by one, the estimated value of the effect size was still within the 95% CI of the original effect size, so the result was relatively stable, as shown in Figure 3.

To further explore the sources of heterogeneity, this study conducted subgroup analysis on the intervention cycle, intervention duration, and intervention methods that may cause the sources of heterogeneity, as shown in Table 3.

**Figure 4.**
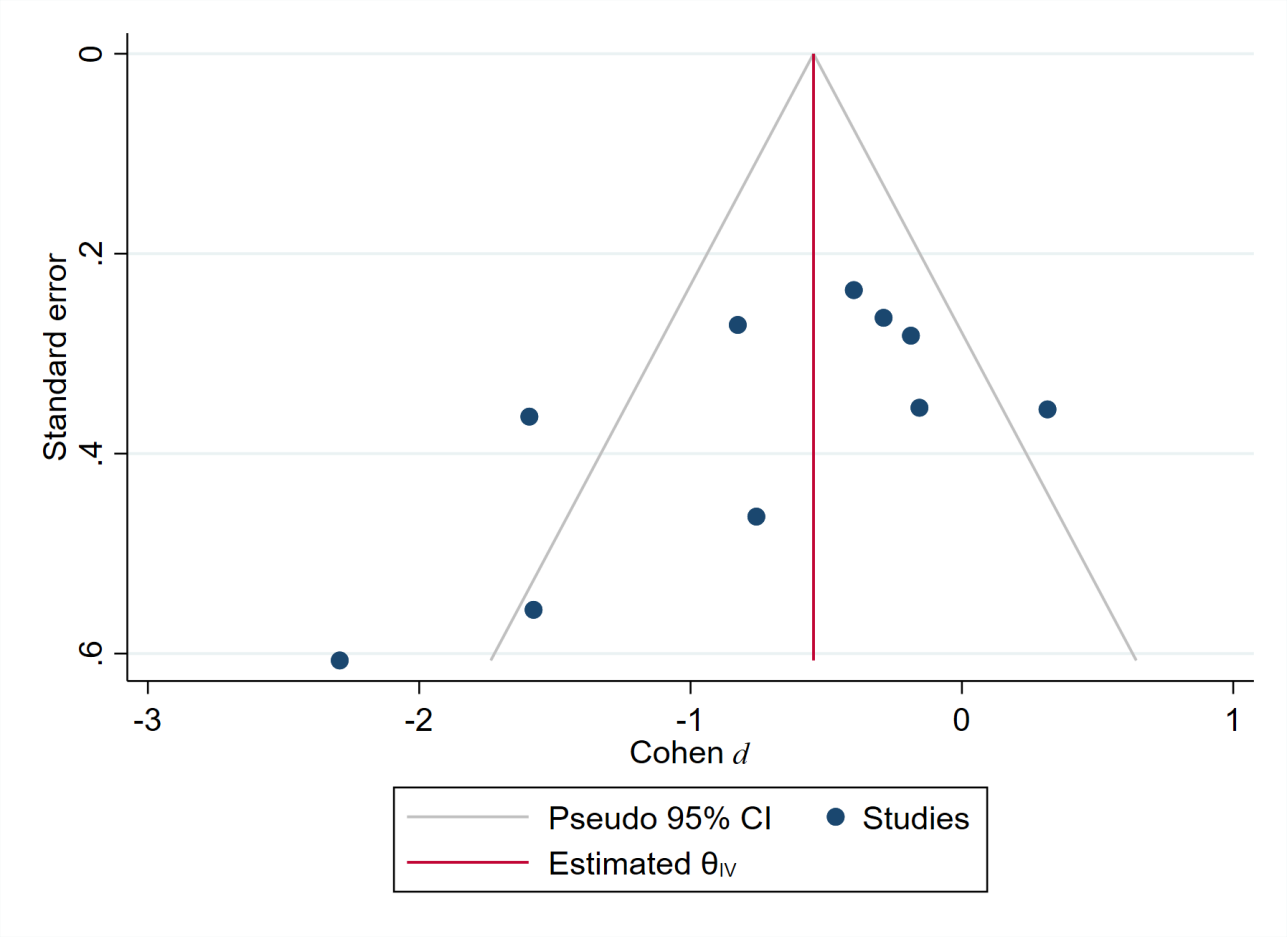
Funnel diagram of the impact of virtual reality technology on patients with attention-deficit/hyperactivity disorder.

The Begg test (Z=−1.25, Prob>| z |=.283) indicated no publication bias, as shown in Figure 4.

**Table 3.** Subgroup analysis of the impact of virtual reality technology on patients with attention-deficit/hyperactivity disorder.

	Q^a^, P	*I* ^2^	Cohen *d* (95% CI)	*P* value
**Intervention cycle (wk）**	1.59, .21			
2‐6		65.07	–1.01 (–1.65 to –0.37)	.002
8‐12		77.26	–0.47 (–1.02 to 0.09)	.10
**Intervention duration (min)**	1.90, .17			
10‐20		71.61	–1.13 (–1.96 to –0.31)	.01
30‐60		75.51	–0.45 (–0.95 to –0.05)	.08
**Intervention methods**	3.92, .05			
Nonimmersive		0	–0.28 (–0.55 to –0.01)	.04
Immersive		78.50	–1.05 (–1.76 to –0.34)	.004
Diagnostic type	4.28, .04			
Formal diagnosis		70.92	–0.44 (–0.85 to –0.03)	.03
Informal diagnosis		50.88	–1.47 (–2.35 to –0.59)	.001

a Q: quotient effect size.

The intervention cycle did not affect the intervention effect (Q=1.59, *P*=.21). The intervention cycle was divided into “2‐6 weeks” and “8‐12 weeks.” For “2-6 weeks” (Cohen *d*=−1.01, 95% CI −1.65 to −0.37; *P*=.002), the difference was statistically significant; for “8‐12 weeks” (Cohen *d*=−0.47, 95% CI −1.02 to 0.09; *P*=.10), the difference was not statistically significant.

The intervention duration did not have a moderating effect on the intervention’s effect (Q=1.90, *P*=.17). The intervention duration was divided into “10‐20 minutes” and “30‐60 minutes.” A Cohen *d* of −1.13 (95% CI −1.96 to −0.31, *P*=.007) indicated a statistically significant difference; a Cohen *d* of −0.45 (95% CI −0.95 to −0.05, *P*=.08) indicated no statistically significant difference.

The intervention methods had a moderating effect on the intervention effect (Q=3.92, *P*=.048). The intervention methods were divided into “nonimmersive” and “immersive” (Cohen *d*=−0.28, 95% CI −0.55 to −0.01; *P*=.04), and the difference was statistically significant (immersive: Cohen *d*=−1.05, 95% CI −1.76 to −0.34; *P*=.004).

The diagnostic type had a moderating effect (Q=4.28, *P*=.04), and was divided into “formal diagnosis” (Cohen *d*=−0.44, 95% CI −0.85 to −0.032; *P*=.03) and “informal diagnosis” (Cohen *d*=−1.47, 95% CI −2.35 to −0.59; *P*=.001), and the differences were statistically significant.

#### Meta-Analysis of VR Technology on the Motor Ability of Children With ADHD

**Figure 5.**
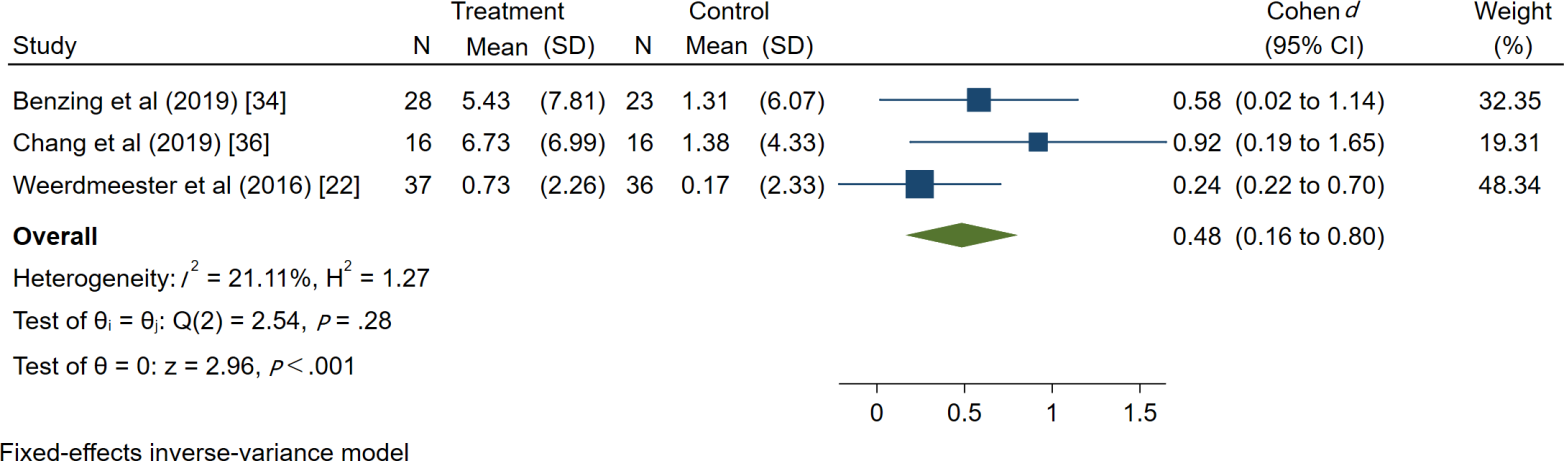
Forest diagram of the impact of virtual reality technology on motor ability in patients with attention-deficit/hyperactivity disorder.

A total of 3 study projects were conducted on 156 patients, exploring the intervention effect of VR technology on the motor ability of patients with ADHD. Heterogeneity results showed that *I*^2^=20.82%, indicating no heterogeneity in our study. Therefore, a fixed effects model was used for effect size combinations. The meta-analysis revealed a Cohen *d* of 0.48 (95% CI 0.16-0.80, *P*<.001), and the difference was statistically significant, indicating that VR technology could improve the motor ability of patients with ADHD, as shown in Figure 5.

As only 3 studies included exercise ability analyses, not exceeding 10 studies, no publication bias was conducted.

### Evaluation of Outcome Quality Level

The GRADEpro software was adopted to evaluate the level of outcome evidence. Since “limitations” and “inconsistency” were downgraded, the quality of attention outcomes was rated as “low.” Due to “limitations” and “imprecision,” the quality level of motor ability outcomes is low, as shown in Table 4.

**Table 4.** Evaluation of outcome quality Level.

Outcome indicators	Randomized controlled trials	Assessment of evidence quality					Number of participants		Relative effect (95% CI)	Quality of evidence
		Limitations	Inconsistency	Indirectness	Imprecision	Publication bias	Experimental group	Control group		
Attention	9	1 grade reduced^a^	1 grade reduced^b^	No	No	No	201	169	–0.68, (–1.12 to −0.24)	Low
Motor ability	3	1 grade reduced^c^	No	No	1 grade reduced^d^	No	81	75	0.48 to (0.16 to 0.80)	Low

a Most studies did not conduct allocation concealment and evaluation blinding, and all included studies were not blinded during conducting research.

b Intergroup homogeneity was high, and the included population was both formally diagnosed and informally diagnosed.

c All included literature was not conducted evaluator blinding.

d There are only three studies included, and the quantity is relatively small.

## Discussion

### Principal Findings

The meta-analysis revealed that VR technology can improve the attention of children with ADHD. However, due to limitations and inconsistencies, the outcome evidence level was low. Moreover, VR technology can also improve the motor ability of children with ADHD, and the level of outcome evidence is also low due to limitations and inaccuracies. Although all the literature included in this study were RCTs, most of them were designed to achieve allocation concealment, evaluation blinding, and researcher blinding, enhancing the additional risk of misleading results and affecting the reliability of the results. Moreover, the included participants may have ADHD symptoms but have not been formally diagnosed, which may lead to inconsistent results. In addition, there are only 3 studies on exercise ability, which also leads to insufficient accuracy in the final results. In future research, on top of using RCTs, efforts should be made to enhance allocation concealment, researcher blinding, and evaluation blinding to improve methodological quality.

The results of this study showed that compared to the control group, VR technology can improve attention in children with ADHD, with an effect size of 0.68. According to Cohen effect size evaluation criteria, VR technology has a moderate effect, which is consistent with previous reports [16,24,25]. This may be due to the immersive, interactive, and imaginative characteristics of VR technology, which allows children with ADHD to attract their attention, maintain their concentration, and improve their attention during long-term education and training. In addition, we found that all 9 studies included serious games, also known as educational games, which are digital games designed to achieve educational, training, or therapeutic goals in an engaging manner, thereby enhancing learners’ or participants’ motivation and involvement [42]. Serious games based on VR technology can improve patients’ perceptual abnormalities and facilitate skill enhancement beyond the intervention targets. For example, Weerdmeester et al [22] designed a VR-based serious game where children role-play as a small dragon through 3 levels: the first focuses on attention and impulse control, the second on hyperactivity, and the third on motor skills. However, this study found moderate heterogeneity among the included literature, which may be caused by the intervention methods, intervention duration, intervention cycle of VR technology, and the diagnostic type of ADHD.

We found that the intervention methods of VR technology had a regulatory effect on the attention of children with ADHD. The “immersive” intervention method has the largest effect size (Cohen *d*=−1.05), belonging to a large effect size, while the “nonimmersive” intervention method has the smallest effect size (Cohen *d*=−0.28), belonging to a small effect size. This may be because “immersive” VR technology is a computer-generated simulation world that needs to block the user’s external environment [43], and typically participants are brought into a virtual space using various head-mounted displays. Due to the limitations of the external environment, immersive VR technology can better replicate the cognitive needs of the real world, and the training results obtained in these environments are better than those obtained in nonimmersive ones [44,45]. Meta-analysis also suggests that immersive VR technology can improve attention in children with ADHD [24]. “Nonimmersive” VR technology, compared to “immersive” intervention method, reduces the patient’s experience but leads to decreased performance [46]. Nevertheless, it still provides multisensory interactive experiences, such as the integration of visual and auditory information, which aids in enhancing pediatric patients’ overall sensory processing abilities and attention control [47]. Goharinejad et al [17] also reported similar results, in that VR and augmented reality can not only effectively evaluate ADHD symptoms but also contribute to the treatment of ADHD symptoms. The diagnostic type has a moderating effect on attention in children with ADHD treated with VR technology. The effect size of “informal diagnosis” is the largest (Cohen *d*=−1.47), belonging to the large effect size category, while the effect size of “formal diagnosis” is the smallest (Cohen *d*=−0.44), belonging to small-to-moderate effect size category. This is partially inconsistent with previous research results. Corrigan et al [24] believed that immersive VR technology had the best effect on improving the overall cognitive function of children with formally diagnosed ADHD, while it did not affect improvements in the cognitive function of children without a formal diagnosis of ADHD. The reasons for the different results may be as follows: (1) previous studies only discussed immersive VR technology, while this study included all types of VR technologies; and (2) previous subgroup analyses focused on the overall cognitive function of children with ADHD, while this study only focused on the subgroup analyses of attention.

We also found that the intervention duration and intervention cycle had no regulatory effect on ADHD children’s attention. Previous studies have obtained similar results; for instance, Corrigan et al [24] carried out a meta-regression analysis to determine the association between the duration of a VR intervention as a variable and the overall cognitive function of ADHD, and found that the duration of intervention did not affect the overall cognitive function of ADHD. Previous studies conducted meta-analyses on children with cerebral palsy and patients with depression, and the results also found that the duration VR interventions did not adjust the effect size of upper limb function and depression [48,49]. However, due to the limited number of included literature, this result should be treated with caution.

The results of this study show that compared with the control group, VR technology can improve the motor ability of children with ADHD, and the effect size was 0.48, which implies small and medium effects. Although there is no systematic review to explore the effect of VR on the motor ability of children with ADHD, experimental studies have obtained similar results. Shema-Shiratzky et al conducted a 6-week, three-times-a-week VR cognitive and motor joint training for children with ADHD, whose results showed that the gait of children with ADHD was improved under the dual task [50]. Benzing and Schmidt [34] conducted a VR game intervention for children with ADHD for 8 weeks, 3 times a week, and 30 minutes each time, and reported that compared to the control group, VR game training could improve the motor ability of children with ADHD. We found that all the included studies on the evaluation of the motor ability of children with ADHD used VR game training, and the completion of VR game training required the participation of children’s physical activities, which can change cerebral blood flow, cause the release of serotonin and brain-derived neurotrophic factor, promotes the increase of catecholamine and proteinase, and then improve the core symptoms of children with ADHD [51,52].

### Strengths and Limitations

#### Strengths

This review followed highly recommended guidelines for PRISMA [27]. Thus, it can be considered a transparent and reproducible review. In comparison with other studies [48], this research includes a greater number of RCTs and, for the first time, uses GRADE (Grading of Recommendations, Assessment, Development, and Evaluation) to assess the graded outcomes of motor skills, thereby enabling researchers to draw more precise conclusions. Furthermore, we explore factors regulating attention in children with ADHD using VR (such as intervention methods, duration, and diagnostic types), providing a theoretical basis for developing VR interventions for children with ADHD. Additionally, the attention outcomes of this study are not subject to publication bias as we searched and included gray literature.

#### Limitations

The limitations of our study include the following. First, a small number of publications was included, among which 9 articles were about attention, and only 3 articles were about motor ability. Second, the clinical heterogeneity mainly manifested in age, and gender, while the heterogeneity in methodology mainly manifested in the decision to conduct allocation concealment, research blinding, and evaluation blinding, thus reducing the reliability of the results. Finally, we did not conduct publication bias due to the limited number of publications included, and there may be small sample bias.

### Implications for Future Research and Practices

In the future development of VR interventions for children with ADHD, it is crucial to fully consider the diagnostic types and intervention methods tailored to the individual characteristics of children with ADHD. VR technology shows promise for both diagnosing and managing ADHD, but efforts should be made to standardize assessment tools to enhance the reliability of outcomes. The application of VR in ADHD is still exploratory and developmental, and its economic costs warrant further discussion. Additionally, large-scale RCTs should be conducted with efforts toward achieving allocation concealment, researcher blinding, and assessor blinding wherever possible, to substantiate the impact of VR on attention and motor function in children with ADHD.

### Conclusions

VR technology can improve attention and motor ability in children with ADHD. The immersive VR technology has the best attention improvement effect for children with an informal diagnosis of ADHD. Future studies should adopt a more rigorous methodological design and include larger populations (eg, multicenter clinical RCTs) to provide further evidence of the beneficial effect of VR technology on children with ADHD.

## Supplementary material

10.2196/56918 Multimedia Appendix 1 PICO framework of the effect of virtual reality technology on ADHD children's attention and motor ability. ADHD: attention-deficit/hyperactivity disorder; PICO: Patient; Intervention; Comparison, Control, or Comparator; and Outcomes.

10.2196/56918 Multimedia Appendix 2 Retrieval strategies of each database.

10.2196/56918 Checklist 1 PRISMA checklist. PRISMA: Preferred Reporting Items for Systematic Reviews and Meta-Analyses.

## Abbreviations

ADHD: attention-deficit/hyperactivity disorder
*DSM*: *Diagnostic and Statistical Manual of Mental Disorders*
GRADE: Grading of Recommendations, Assessment, Development, and Evaluation
*ICD-10*: *International Statistical Classification of Diseases, Tenth Revision*
ITT: intention-to-treat
PICO: Patient; Intervention; Comparison, Control, or Comparator; and Outcomes
PRISMA: Preferred Reporting Items for Systematic Reviews and Meta-Analyses
RCT: randomized controlled trial
VR: virtual reality
