# Supplementary material for: Effect of Virtual Reality Technology on Attention and Motor Ability in Children With Attention-Deficit/Hyperactivity Disorder: Systematic Review and Meta-Analysis
Source: JMIR Serious Games. 2024 Nov 27;12:e56918. doi: 10.2196/56918 (PMC11612531; doi:10.2196/56918)
Supplement: Multimedia Appendix 1 [file games-v12-e56918-s001.docx]

| Population | Intervention | Comparison | Outcome | Study design |
| --- | --- | --- | --- | --- |
| Children with formally diagnosed ADHD;  Children with informally diagnosed ADHD. | Intervention site: Hospital,  School.  Intervention staff:  Psychologist,  Healthcare personnel,  Therapist.  Type of intervention:  Intervention VR-based technology.  Intervention prescription:  means of intervention,  Intervention frequency,  Intervention duration,  Intervention cycle | No VR technology intervention | Attention  Motor Ability | Randomized Controlled Trial |
