# Supplementary material for: Effect of Virtual Reality Technology on Attention and Motor Ability in Children With Attention-Deficit/Hyperactivity Disorder: Systematic Review and Meta-Analysis
Source: JMIR Serious Games. 2024 Nov 27;12:e56918. doi: 10.2196/56918 (PMC11612531; doi:10.2196/56918)
Supplement: Multimedia Appendix 2 [file games-v12-e56918-s002.docx]

| Database | Search Strategy |
| --- | --- |
| Cochrane  and  Pubmed | #1 “virtual reality”[Mesh] OR “VR” [Title/Abstract] OR “Reality, Virtual” [Title/Abstract] OR “virtual reality” [Title/Abstract] OR “game based virtual reality” [Title/Abstract] OR “computer based virtual reality” [Title/Abstract] OR “active video games” [Title/Abstract] OR “virtual game” [Title/Abstract] OR “virtual environment” [Title/Abstract]  #2 “Attention Deficit Disorder with Hyperactivity” [Mesh] OR “Attention Deficit Disorder with Hyperactivity” [Title/Abstract] OR “attention deficit hyperactivity disorder” [Title/Abstract] OR “ADHD” [Title/Abstract] OR “Attention Deficit Disorder” [Title/Abstract] OR “ADDH” [Title/Abstract] OR “Hyperkinetic Syndrome” [Title/Abstract]  #3 #1 AND #2 |
| Embase | 1 “virtual reality” [exp] OR “VR”[ab,ti]OR “Reality, Virtual” [ab,ti] OR “virtual reality” [ab,ti] OR “game based virtual reality” [ab,ti] OR “computer based virtual reality” [ab,ti] OR “active video games” [ab,ti] OR “virtual game” [ab,ti] OR “virtual environment” [ab,ti]  #2 “attention deficit hyperactivity disorder” [exp] OR “Attention Deficit Disorder with Hyperactivity” [ab,ti] OR  “attention deficit hyperactivity disorder” [ab,ti] OR “ADHD”[ab,ti] OR “Attention Deficit Disorder” [ab,ti] OR “ADDH” [ab,ti] OR “Hyperkinetic Syndrome” [ab,ti]  #3 #1 AND #2 |
| Web of  Science | #1 TS=（ “virtual reality” OR “VR” OR “Reality, Virtual” OR “game based virtual reality” OR “computer based virtual reality” OR “active video games” OR “virtual game” OR “virtual environment” ）  #2 TS=（ “attention deficit hyperactivity disorder” OR “Attention Deficit Disorder with Hyperactivity” OR “ADHD” OR “Attention Deficit Disorder” OR “ADDH” OR “Hyperkinetic Syndrome”  #3 #1 AND #2 |
